# Does the Serum Testosterone Level Have a Relation to Coronary Artery Disease in Elderly Men?

**DOI:** 10.1155/2011/791765

**Published:** 2011-05-23

**Authors:** Mohamed A. Helaly, Eid Daoud, Noha El-Mashad

**Affiliations:** ^1^Department of Internal Medicine, Specialized Medical Hospital, Faculty of Medicine, Mansoura University, B.O. Pox 35516, Al-Gomhoria Street, Mansoura, Egypt; ^2^Department of Cardiology, Faculty of Medicine, Mansoura University, B.O. Pox 35516, Mansoura, Egypt; ^3^Department of Clinical Pathology, Faculty of Medicine, Mansoura University, B.O. Pox 35516, Mansoura, Egypt

## Abstract

*Background*. The low serum level of testosterone in the elderly subjects may contribute to coronary artery disease (CAD). Our aim is to study serum levels of free testosterone in elderly men with CAD. *Subjects and Methods*. This study was conducted on 100 elderly males with CAD, one half of them was presented with ACS (with mean age 69.6 ± 2.44 year), and the other half was presented with stable CAD (with mean age 69.42 ± 2.14 year), in addition to 50 apparently healthy elderly males (with mean age 69.06 ± 1.64 year) as a control group. We detected the levels of serum free testosterone, cortisol, fibrinogen, plasminogen activator inhibitor-1(PAI-1), high sensitive C-reactive protein(hsCRP), interleukin-6(IL-6). *Results*. Cases with CAD had significant lower values of free testosterone and HDL-c, but they had significant higher values of cortisol, fibrinogen, PAI-1, IL-6, hsCRP, in comparison to control group. Cases with ACS had significant higher values of cortisol, hsCRP, IL-6, fibrinogen, PAI-1, total cholesterol and BMI more than those with stable CAD. The free testosterone had significant negative correlation with fibrinogen, PAI-1, hsCRP and IL-6 in both groups of patients. *Conclusion*. The lower value of serum free testosterone in elderly male subjects may contribute to CAD.

## 1. Introduction and Aim of the Work

Coronary artery disease (CAD) is common in old people and accounts for about half of all deaths in those people [1]. Phillips et al. [2] reported that low total and free testosterone levels were inversely linked to coronary artery disease, even after adjusting for age and adiposity. This idea was supported by another study, in which men with angiographically proven CAD have lower levels of testosterone than those of control subjects, and the testosterone levels were negatively correlated to the degree of coronary involvement [3]. Moreover, lower testosterone levels predict incident stroke and transient ischemic attacks in older men [4]. Few studies were done for the association of testosterone level with CAD in elderly men.

The aim of this work is to study the levels of serum free testosterone in elderly patients with CAD (both those presenting with acute coronary syndrome and others with stable CAD) and the correlation of free testosterone with levels of serum cortisol, fibrinogen, PAI-1, hsCRP, and IL-6 in those patients.

## 2. Subjects and Methods

A total of 100 elderly male patients with CAD with a mean age of >65 years were taken from the Cardiology and Geriatric Units (age recruitment criteria, the man was >65), Specialized Medical Hospital, Mansoura University in the period between October 2009 to June 2010. The patients were then subsequently divided according to the clinical presentation of CAD into *group 1* which consists of 50 patients with acute coronary syndrome; (21 of them had ST elevation myocardial infarction (STEMI), 14 of them had non-ST elevation myocardial infarction (NSTEMI), and 15 had unstable angina), and *group 2* which consists of 50 patients with stable CAD. This is in addition to 50 apparently healthy elderly subjects; they are nonsmokers and had no risk factors for CAD as a control group (*group 3*). All cases and control subjects were subjected to thorough history taking with special stress on symptoms suggestive of CAD and/or symptoms of ACS and history of previous cardiac interventions done for the patient, in addition to full clinical examination, 12-lead ECG, and echocardiography. Cases with stable CAD were diagnosed after coronary angiography. Unstable angina was diagnosed according to EKD only if the patient had one or more of the following 3 principal presentations: (1) rest angina (usually lasting for >20 minutes), (2) new onset angina (<2 months previously), (3) Crescendo angina (increasing in intensity, duration, frequency, or any combination of these factors [5] with or without ECG changes in the form of ST-segment depression, transient ST-segment elevation, T-wave inversion, or some combination of these changes [6]).

Patients with acute myocardial infarction were diagnosed if they had 2 of the following: (1) chest pain of >30 minutes, (2) ≥2-fold increase in serum creatine phosphokinase (CK) with elevation of MB isoform ≥10%, (3) persistent ischemic ECG changes: evolution of pathologic Q-waves (≥0.04 second), or ≥1 mm ST-segment elevation in at least 2 contiguous leads or new onset left bundle branch block (in cases of STEMI), or ≥0.5 mm ST-depression or definite T-wave inversion (in cases of NSTEMI) [7], and unfortunately troponins not done. 

10 mL of overnight fasting blood samples were drawn for the determination of free testosterone, cortisol, fibrinogen, plasminogen activator inhibitor-1 (PAI-1), IL-6, hsCRP, total cholesterol, triglyceride (TG), low-density lipoprotein-cholesterol (LDL-c), high-density lipoprotein-cholesterol (HDL-c), creatinine, creatine phosphokinase (CPK), and MB fraction.

Free serum testosterone and serum cortisol were determined by radioimmunoassay [8, 9].

PAI-1 level was detected by radioimmunoassay [10].

Fibrinogen level was determined by clotting method (FIBRI-PREST2): 1 : 10 dilution of plasma was tested with buffer diluents (0.1 mL of plasma plus 0.9 mL of buffer), then dilutions in an ice bath were placed. Dilutions were stable for up to 4 hours. Clotting time was determined for each sample and duplicated. The average duplicated times for each dilution were obtained. the fibrinogen concentration was obtained from a calibration curve [11].

IL-6 was detected by goat antirabbit antibody immunoassay: goat antirabbit antibodies are used to capture a specific IL-6 complex in each sample consisting of IL-6 antibody, biotinylated IL-6, and sample/standard. The biotinylated IL-6 conjugate (competitive ligand) and sample or standard compete for IL-6-specific antibody-binding sites. Therefore, as the concentration of IL-6 in the sample increases, the amount of biotinylated IL-6 captured by the antibody decreases. The assay is visualized using a streptavidin alkaline phosphatase conjugate and a chromogenic substrate reaction. The amount of IL-6 detected in each sample is compared to an IL-6 standard curve which demonstrates an inverse relationship between optical density (OD) and cytokine concentration, that is, the higher the OD, the lower the IL-6 concentration in the sample.

hsCRP was detected using an enzymatically amplified “two-step” sandwich type immunoassay, standards, and controls, and unknown samples were incubated in microtitration plates which have been coated with anti-hs-CRP antibody. After incubation and washing, wells were treated with another anti-hs-CRP detection antibody labelled with enzyme HRP. After a second incubation, wells were incubated with substrate FMB. An acidic stopping solution is then added, and the degree of enzymatic turnover of the substrate is determined by the measurement at 450 nm. The absorbance measured is directly proportional to the concentration of hs-CRP present. Results of these samples were multiplied by 500 to correct for the additional dilution.

### 2.1. Exclusion Criteria

1. Patients with major organ failure (heart, respiratory, liver, or renal failure).
2. Patients with cancer prostate, prostatectomy, castration or those receiving androgen deprivation therapy.
3. Patients receiving androgens, steroids, or hypolipidemic medications.
4. Patients with diabetes mellitus or other endocrine disorders.
5. Smoker patients and controls were also excluded from the study to avoid the possible interaction between smoking and testosterone serum level.
6. Those with active infection or autoimmune diseases were excluded.

### 2.2. Statistical Analysis

The statistical analysis of the data was done using SPSS (Statistical Package of Social Science) Program version 10. The data was presented in the form of mean ± standard deviation for quantitative data and frequency and proportion for qualitative data. For comparing statistical significance between 2 groups, *t*-test was used. For qualitative data, chi-square test was used. Pearson correlation coefficient was used to study correlation between variables. Significance was considered when the *P* value was less than  .05 at a confidence interval of 95%.

## 3. Results

Cases with CAD (group 1 + 2) have significant higher values of BMI, systolic blood pressure (SBP), diastolic blood pressure (DBP), fasting plasma glucose (FPG), serum creatinine, total cholesterol, triglyceride (TG), low-density lipoprotein-cholesterol (LDL-c), serum cortisol, high sensitive C-reactive protein (hsCRP), interleukin-6 (IL-6), fibrinogen, and plasminogen activator inhibitor-1 (PAI-1), but they have lower values of free testosterone and HDL-c in comparison to control group as shown in Table 1.

Cases with acute coronary syndrome (group 1) have significant higher values of BMI, total cholesterol, cortisol, hsCRP, IL-6, fibrinogen, and PAI-1 more than those with stable CAD (group 2), while there is no significant difference as regards free testosterone between both groups as shown in Table 2.

**Figure 1:**
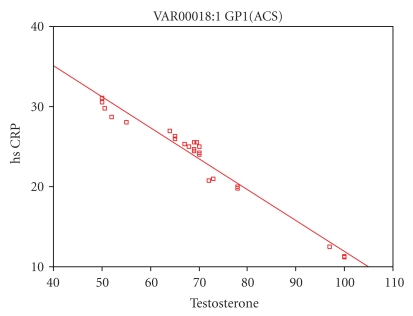
Correlation of free testosterone with hsCRP in patients with ACS (group 1).

Free testosterone in cases with ACS has significant negative correlation with BMI, SBP, DBP, FPG, serum creatinine, total cholesterol, TG, cortisol, hsCRP, IL-6, fibrinogen, and PAI-1 as shown in Table 3 and in Figure 1.

**Figure 2:**
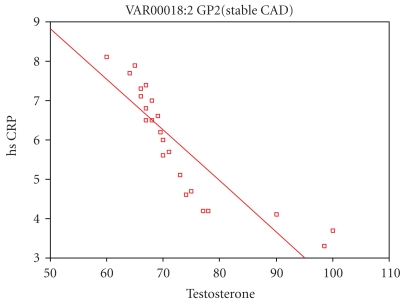
Correlation of free testosterone with hsCRP in patients with stable CAD (group 2).

The free testosterone also in cases with stable CAD has significant negative correlation with BMI, serum creatinine, total cholesterol, LDL-c, hsCRP, IL-6, fibrinogen, and PAI-1 as shown in Table 3 and in Figure 2.

## 4. Discussion

Previous studies had suggested that hypogonadism and androgen deficiency were linked to CAD in men [2, 12]. Although our patients in this study had no hypogonadism, they had a significant lower serum free testosterone level in comparison to control subjects, but there is no significant difference regarding it between cases with ACS and those with stable CAD. This agree with Liu et al. [13] who suggested that testosterone levels were consistently lower in men with CAD. The pathophysiology of cardiovascular risk of low testosterone serum level may be related to increased fat mass [14]. A host of previous studies have suggested that reduced testosterone levels were associated with increased total cholesterol and LDL-c [15–17] and increased TG and reduced HDL-c [18]. In other studies, the systolic and diastolic blood pressures have been shown to be inversely correlated with testosterone level [19]. These studies agree with the results in our study in which the cases with CAD had significant higher values of BMI, SBP, DBP, FPG, total cholesterol, TG, and LDL-c more than control subjects, but they had a significant lower values of HDL-c. Moreover, the cases with ACS had significant higher values of BMI, total cholesterol, and LDL-c more than those with stable CAD. Dobrzycki et al. [20] showed that patients with at least a 50% lesion of at least one coronary vessel had significantly elevated systolic and diastolic blood pressure, fibrinogen, and TG levels, and reduction in ejection fraction, total and free testosterone, and HDL-c. 

The morning serum cortisol level was significantly higher in cases more than control subjects, and moreover, its level was significantly higher also in those with ACS more than those with stable CAD possibly related to stress induced by acute coronary event. In the Caperhilly Study (South Wales), Smith et al. [21] followed up more than 2500 men for a mean of 16.5 years and found that the cortisol/testosterone ratio had a specific association to ischemic heart disease, possibly related to chronic illness, mediated through insulin resistance. 

The contribution of low testosterone level to the development of CAD is not only through its deleterious effects on blood pressure, glucose tolerance, and plasma lipid, but also because it increases blood coagulability via increasing fibrinogen, plasminogen activator inhibitor-1, and factor 7 [22]. In our study, fibrinogen and PAI-1serum levels were significantly higher in cases with CAD more than in control group, and moreover, their values were significantly higher in patients with ACS more than those with stable CAD. 

The levels of serum hsCRP and IL-6 in cases with CAD in our study were significantly higher in comparison to the control group. Their levels, moreover, were also higher in cases with ACS than in those with stable CAD which may indicate augmented inflammatory state in which low testosterone level have a role. These results go with the previous studies like those of Yialamas et al. [23] and Maggio et al. [24] which found that androgen deficiency is associated with increased production of inflammatory cytokines. These associations were further validated by clinical trials showing improvement in lipid profile and reduction in inflammatory cytokines with testosterone treatment [25].

The free testosterone serum level in patients with ACS in our study had significant negative correlation with BMI, SBP, DBP, FPG, total cholesterol, TG, cortisol, fibrinogen, PAI-1, hsCRP, and IL-6. Similarly, free testosterone serum level had significant negative correlation with BMI, total cholesterol, LDL-c, fibrinogen, PAI-1, hsCRP, and IL-6 in cases with stable CAD. Dobs et al. [26] investigated hypogonadal men who were treated for one year with testosterone patch to set up simple correlations. The authors found that BMI has strong negative correlation with testosterone.

## 5. Summary and Conclusion

Coronary artery disease (CAD) in elderly men is associated with low serum testosterone level even within normal range, and this may contribute to its pathogenesis thorough increase in blood pressure and glucose levels, dyslipidaemia, visceral obesity, endothelial dysfunction, and increase in pro-inflammatory cytokines, and this may explain the high incidence of CAD in elderly men. Whether testosterone supplementation for elderly men is beneficial in primary or secondary prevention of CAD in them needs further prospective multicenter trial done on a large number of population.

## Figures and Tables

**Table 1:** Comparison of demographic, clinical, and laboratory data of cases versus control group.

	Cases with CAD	Control (no CAD)	Significance
	Mean ± S.D.	Mean ± S.D.	(*P* value)
Age (years)	69.51 ± 2.29	69.06 ± 1.64	.17
BMI (kg/m^2^)	27.99 ± 3.08	23.10 ± 0.71	.0001
SBP (mmHg)	146.70 ± 9.02	125.20 ± 7.28	.0001
DBP (mmHg)	91.76 ± 9.36	75.20 ± 7.14	.0001
FPG (mg/dL)	87.95 ± 12.71	79.08 ± 5.49	.0001
Serum creatinine (mg/dL)	1.09 ± 0.19	1.02 ± 0.09	.004
Total cholesterol (mg/dL)	209.53 ± 36.20	132.84 ± 10.15	.0001
TG (mg/dL)	161.61 ± 53.27	80.24 ± 7.09	.0001
LDL-c (mg/dL)	142.82 ± 35.84	76.88 ± 7.49	.0001
HDL-c (mg/dL)	37.96 ± 4.97	47.66 ± 5.06	.0001
Cortisol (nmol/L)	359.10 ± 137.47	220.14 ± 45.01	.0001
Free testosterone (pg/mL)	70.75 ± 11.95	164.60 ± 29.56	.0001
hs CRP (mg/L)	14.81 ± 9.74	1.45 ± 0.60	.0001
IL-6 (ng/L)	7.33 ± 3.22	1.94 ± 0.59	.0001
Fibrinogen (g/L)	5.66 ± 1.19	2.93 ± 0.64	.0001
PAI-1 (ng/mL)	56.05 ± 13.56	24.24 ± 9.37	.0001

BMI: body mass index, DBP: diastolic blood pressure, FPG: fasting plasma glucose, HDL-c: high-density lipoprotein-cholesterol, hsCRP: high sensitive C-reactive protein, IL-6: interleukin-6, LDL-c: low-density lipoprotein-cholesterol, PAI-1: plasminogen activator inhibitor-1, Tg: triglycerides.

**Table 2:** Comparison of group 1 (cases with ACS) versus group 2 (cases with stable CAD).

	Group 1	Group 2	Significance
	Mean ± S.D.	Mean ± S.D.	(*P*-value)
Age (years)	69.60 ± 2.44	69.42 ± 2.14	.697
BMI (kg/m^2^)	29.11 ± 3.41	26.86 ± 2.23	.0001
SBP (mmHg)	148.60 ± 9.53	144.80 ± 8.14	.035
DBP (mmHg)	91.52 ± 11.76	92.00 ± 6.22	.799
FPG (mg/dL)	85.98 ± 14.33	89.92 ± 10.65	.122
Serum creatinine (mg/dL)	1.07 ± 0.23	1.12 ± 0.13	.175
Total cholesterol (mg/dL)	224.22 ± 33.25	194.84 ± 33.20	.0001
TG (mg/dL)	171.80 ± 72.60	151.42 ± 15.84	.058
LDL-c (mg/dL)	151.98 ± 39.97	133.66 ± 28.74	.010
HDL-c (mg/dL)	37.54 ± 6.34	38.38 ± 3.07	.402
Cortisol (nmol/L)	487.36 ± 25.52	230.84 ± 62.91	.0001
Free testosterone (pg/ mL)	69.68 ± 14.34	71.82 ± 8.98	.374
hs CRP (mg/L)	23.61 ± 5.64	6.01 ± 1.33	.0001
IL-6 (ng/L)	8.69 ± 3.66	5.97 ± 1.94	.0001
Fibrinogen (g/L)	6.11 ± 1.27	5.20 ± 0.91	.0001
PAI-1 (ng/mL)	63.70 ± 10.22	48.40 ± 12.15	.0001

**Table 3:** Correlation of free testosterone with other data in group 1 and group 2.

	Free testosterone in group 1		Free testosterone in group 2	
	Pearson correlation (*V*)	Significance (*P* value)	Pearson correlation (*V*)	Significance (*P* value)
Age	−0.335	.017	−0.361	.010
BMI	−0.765	.0001	−0.821	.0001
SBP	−0.601	.0001	−0.298	.036
DBP	−0.457	.001	0.038	.796
FPG	−0.482	.0001	−0.125	.388
Serum creatinine	−0.543	.0001	−0.559	.0001
Total cholesterol	−0.546	.0001	−0.431	.002
TG	−0.419	.002	−0.118	.414
LDL-c	−0.091	.531	−0.588	.0001
HDL-c	0.164	.256	0.114	.430
Cortisol	−0.804	.0001	−0.157	.275
hs CRP	−0.982	.0001	−0.871	.0001
IL-6	−0.781	.0001	−0.726	.0001
Fibrinogen	−0.745	.0001	−0.912	.0001
PAI-1	−0.682	.0001	−0.863	.0001
